# Mitocryptide-2: Identification of Its Minimum Structure for Specific Activation of FPR2–Possible Receptor Switching from FPR2 to FPR1 by Its Physiological C-terminal Cleavages

**DOI:** 10.3390/ijms22084084

**Published:** 2021-04-15

**Authors:** Takayuki Marutani, Kodai Nishino, Tomoyuki Miyaji, Keisuke Kamada, Koji Ohura, Yoshiaki Kiso, Hidehito Mukai

**Affiliations:** Laboratory of Peptide Science, Graduate School of Bio-Science, Nagahama Institute of Bio-Science and Technology, Nagahama, Shiga 526-0829, Japan; b210052@nagahama-i-bio.ac.jp (T.M.); b112116@m.nagahama-i-bio.ac.jp (K.N.); b116169@m.nagahama-i-bio.ac.jp (T.M.); b116046@m.nagahama-i-bio.ac.jp (K.K.); b116028@m.nagahama-i-bio.ac.jp (K.O.); y_kiso@nagahama-i-bio.ac.jp (Y.K.)

**Keywords:** cryptide, mitocryptide, N-formylated peptide, neutrophil, formyl peptide receptor 1, formyl peptide receptor 2, inflammation

## Abstract

Mitocryptides are a novel family of endogenous neutrophil-activating peptides originating from various mitochondrial proteins. Mitocryptide-2 (MCT-2) is one of such neutrophil-activating peptides, and is produced as an N-formylated pentadecapeptide from mitochondrial cytochrome *b*. Although MCT-2 is a specific endogenous ligand for formyl peptide receptor 2 (FPR2), the chemical structure within MCT-2 that is responsible for FPR2 activation is still obscure. Here, we demonstrate that the N-terminal heptapeptide structure of MCT-2 with an N-formyl group is the minimum structure that specifically activates FPR2. Moreover, the receptor molecule for MCT-2 is suggested to be shifted from FPR2 to its homolog formyl peptide receptor 1 (FPR1) by the physiological cleavages of its C-terminus. Indeed, N-terminal derivatives of MCT-2 with seven amino acid residues or longer caused an increase of intracellular free Ca^2+^ concentration in HEK-293 cells expressing FPR2, but not in those expressing FPR1. Those MCT-2 derivatives also induced β-hexosaminidase secretion in neutrophilic/granulocytic differentiated HL-60 cells via FPR2 activation. In contrast, MCT-2(1–4), an N-terminal tetrapeptide of MCT-2, specifically activated FPR1 to promote those functions. Moreover, MCT-2 was degraded in serum to produce MCT-2(1–4) over time. These findings suggest that MCT-2 is a novel critical factor that not only initiates innate immunity via the specific activation of FPR2, but also promotes delayed responses by the activation of FPR1, which may include resolution and tissue regeneration. The present results also strongly support the necessity of considering the exact chemical structures of activating factors for the investigation of innate immune responses.

## 1. Introduction

Neutrophils are a type of leukocyte that are involved in the innate defense system [1,2,3]. Neutrophils comprise the majority of peripheral leukocytes and normally exist in the bloodstream to monitor for infection and tissue damage. When tissue injury occurs due to bacterial infections or internal tissue damage, neutrophils immediately migrate to and infiltrate the injury site. The infiltrated neutrophils are then activated and exert their functions, including superoxide production and phagocytosis of invading bacterial components and toxic substances.

Bacterial N-formylated proteins and peptides, including formyl-Met-Leu-Phe (fMLF) [4,5], complement related factors such as component 5a [6,7], and some chemokines such as IL-8 [8,9] chemoattract and activate neutrophils to promote inflammatory reactions. Moreover, various mitochondrial-derived peptides that also activate neutrophils were recently identified in mammalian tissues [10,11,12,13,14]. Namely, we isolated and identified the novel neutrophil-activating peptides mitocryptide-1, mitocryptide-2 (MCT-2), and mitocryptide-CYC, which are cleaved from mitochondrial cytochrome c oxidase subunit VIII, cytochrome *b*, and cytochrome *c*, respectively, from porcine heart [10,11,12,13]. In addition, we found the possible presence of many mitocryptides that were derived from various mitochondrial proteins to induce the migration and activation of neutrophils [10,14].

Recently, there has also been interest in the roles of mitochondria and their derived substances in innate immunity. Indeed, various tissues damaged by trauma or burns release mitochondria and their contents into the bloodstream [15,16,17,18,19,20,21]. Mitochondria and their contents are also released from a variety of cells damaged by bacterial infection [21,22,23,24,25]. In addition, it is suggested that mitochondria and their derived substances are leaked into the cerebrospinal fluid from damaged tissues in patients with Alzheimer’s disease [26,27]. These released mitochondria and their derived factors, which are called mitochondrial damage-associated molecular patterns (mtDAMPs), promote various innate immune responses, including induction of neutrophil and macrophage migration and activation [15,17,21,28,29]. MtDAMPs also stimulate the secretion of inflammatory mediators from mast cells [30].

N-formylated peptides may constitute one family of the activating factors in mtDAMPs because inhibitors against the functions of N-formylated peptides prevent the activity of mtDAMPs [15,17,28]. Specifically, as 13 proteins are encoded in mitochondrial DNA and are translated in mitochondria as N-formylated forms [31,32], these protein-derived N-formylated peptides may exist in mtDAMPs, and are thought to cause proinflammatory responses including neutrophil infiltration and activation. As MCT-2 is the only endogenous N-formylated peptide that has been isolated from mammalian tissues so far and its complete chemical structure has been determined, it is a leading candidate for such activating factors in mtDAMPs.

Formyl peptide receptor 1 (FPR1, formerly referred to as formyl peptide receptor) and formyl peptide receptor 2 (FPR2, formerly referred to as formyl peptide receptor-like 1) recognize not only N-formylated proteins and peptides derived from bacteria but also endogenous N-formylated peptide MCT-2 and putative peptides derived from mitochondrial N-formylated proteins [33,34,35,36,37,38]. These receptors are expressed mainly by inflammatory immune cells such as neutrophils and macrophages, and their activation induces not only the infiltration of neutrophils and macrophages into injury sites but also the phagocytosis of toxic debris, superoxide generation, and the production of inflammatory cytokines [35,37,38]. FPR2 is also expressed by endothelial cells, and its activation causes an increase in vascular permeability to promote further neutrophil infiltration from the bloodstream to injury sites during the initial stage of inflammation [38,39]. Moreover, FPR1 is proposed to be involved in the processes of resolution and wound healing/tissue regeneration. Indeed, FPR1 activation induces the proliferation of various cells, including epithelial cells and hepatocytes, to promote tissue regeneration [40,41,42,43,44]. In this way, FPR1 and FPR2 are thought to participate throughout the initiation of the proinflammatory response by tissue damage, healing, and regeneration.

However, it is unknown how FPR2 and FPR1 recognize various endogenous N-formylated peptides, including MCT-2. FPR2 specifically recognizes MCT-2 and its N-terminal derivatives longer than ten amino acid residues to activate neutrophils [36,45]. Putative endogenous N-formylated peptides derived from mitochondrial DNA-encoded proteins activate FPR2 and/or FPR1, i.e., NADH dehydrogenase subunit 4(1–20) and NADH dehydrogenase subunit 5(1–28) specifically activate FPR2, whereas NADH dehydrogenase subunit 6(1–6) specifically activates FPR1, and cytochrome *c* oxidase subunit I(1–13) activates FPR1 and FPR2 [46]. However, the chemical structure within endogenous N-formylated peptide MCT-2 that is specifically recognized by FPR2 as well as those within endogenous N-formylated peptides recognized by FPR1 or FPR2 are unclear, although the N-formyl group of those peptides is known to be essential for recognition by FPR2 and FPR1 [45].

In this study, we investigated the structure–activity relationships of MCT-2 and its derivatives to elucidate how FPR2 recognizes MCT-2. We also explored the time-dependent alterations of the molecular forms of MCT-2 in serum and attempted to elucidate the meaning of these alterations in innate immune responses.

## 2. Results

### 2.1. Effects of MCT-2(1–15) and Its Derivatives on [Ca^2+^]_i_ in HEK-293 Cells Stably Expressing FPR1 or FPR2

**Figure 1:**
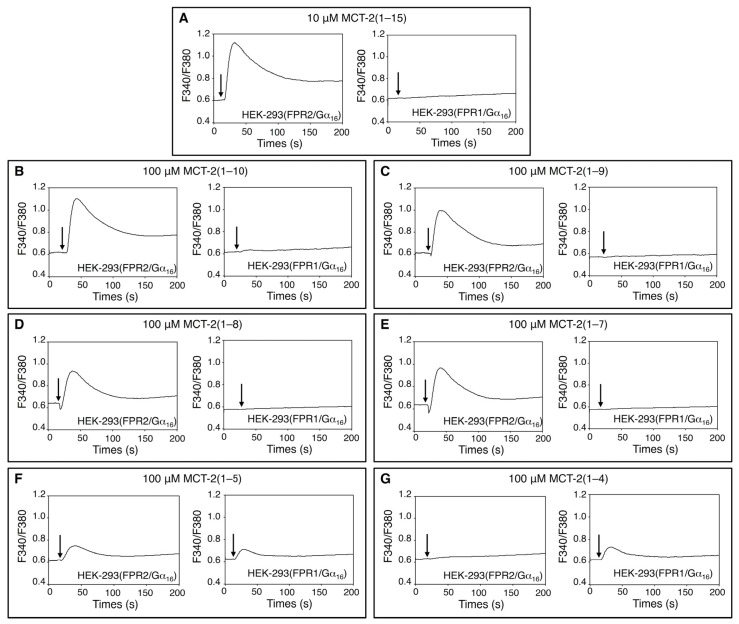
Changes in [Ca^2+^]_i_ in HEK-293 cells stably expressing FPR1 (formyl peptide receptor 1) or FPR2 (formyl peptide receptor 2) induced by MCT-2 (Mitocryptide-2)(1–15) and its derivatives. Fura-2-loaded cells were stimulated with 10 μM MCT-2(1–15) (**A**) or 100 μM MCT-2(1–10) (**B**), MCT-2(1–9) (**C**), MCT-2(1–8) (**D**), MCT-2(1–7) (**E**), MCT-2(1–5) (**F**), and MCT-2(1–4) (**G**). The changes in the fluorescence ratio of Fura-2 (excitation wavelengths, 340 nm and 380 nm; emission wavelength, 500 nm) were recorded by a fluorometer CAF-100. Horizontal axes show the times after stimulation. Vertical axes depict the fluorescence ratios at excitation wavelengths of 340 nm and 380 nm. Arrows indicate the timing of peptide administration.

An endogenous pentadecapeptide, MCT-2 [MCT-2(1–15)], specifically binds to and activates FPR2, but it neither interacts with nor activates FPR1 [36]. In addition, MCT-2(1–10) or N-terminal derivatives longer than it specifically activate FPR2 to cause β-arrestin recruitment and superoxide production [45]. However, the precise structure within MCT-2(1–15) that is responsible for the specific activation of FPR2 is not known. Here, increases in the concentration of intracellular free Ca^2+^ ([Ca^2+^]_i_) promoted by MCT-2(1–15) and its derivatives were assessed in HEK-293 cells stably expressing FPR1 or FPR2 (Figure 1). The Gα_16_ type of G protein was also stably co-expressed in these cells because this protein interacts with various G protein-coupled receptors and effectively induces an agonist-promoted increase of [Ca^2+^]_i_ [47]. As a result, 100 μM MCT-2(1–10), MCT-2(1–9), MCT-2(1–8), and MCT-2(1–7) as well as 10 μM MCT-2(1–15) induced an increase of [Ca^2+^]_i_ in HEK-293 cells expressing FPR2, but not in FPR1-expressing cells (Figure 1A–E). In contrast, 100 μM MCT-2(1–5) induced an increase of [Ca^2+^]_i_ in FPR1- and FPR2-expressing cells (Figure 1F). Moreover, 100 μM MCT-2(1–4) promoted an increase of [Ca^2+^]_i_ in FPR1-expressing cells, but not in FPR2-expressing cells (Figure 1G). In addition, stimulation with MCT-2(1–6) did not induce an increase of [Ca^2+^]_i_ in FPR2- or FPR1-expressing cells, even at a concentration of 100 μM (data not shown). These results demonstrate that the N-terminal derivative MCT-2(1–7) and its longer derivatives specifically activate FPR2, in contrast with MCT-2(1–4), which specifically activates FPR1.

### 2.2. Effects of C- or N-Terminal Truncations of MCT-2(1–15) on β-Hexosaminidase Release from Neutrophilic/Granulocytic Differentiated HL-60 Cells

**Figure 2:**
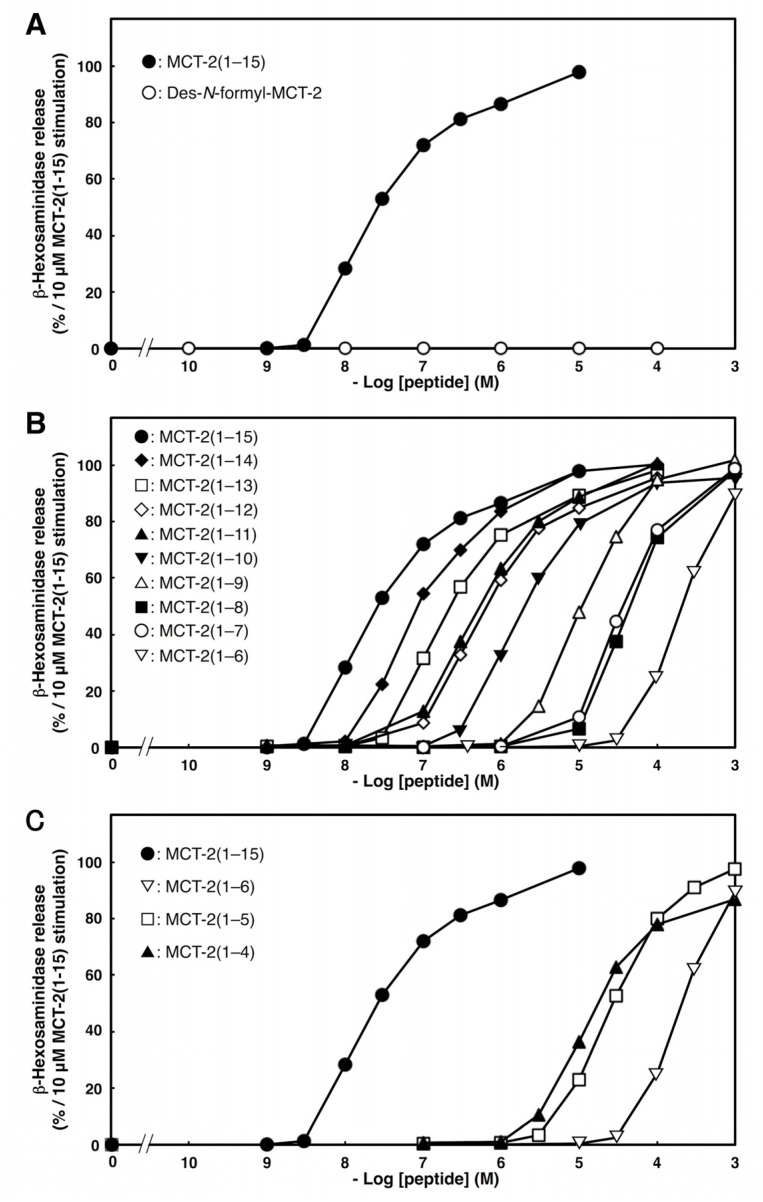
Effects of N-terminal (**A**) or C-terminal (**B**,**C**) truncations of MCT-2(1–15) on β-hexosaminidase release by differentiated HL-60 cells. The differentiated HL-60 cells were stimulated by MCT-2(1–15) or its derivatives at 37 °C for 10 min, and the amount of the released β-hexosaminidase was quantified as described in “Materials and Methods”. The ability of each peptide to induce β-hexosaminidase release is expressed as a percentage of enzyme secretion promoted by 10 μM MCT-2(1–15). Data are expressed as the mean ± SE of four to six independent experiments.

The effects of N- or C-terminal truncations of MCT-2 on β-hexosaminidase released from neutrophilic/granulocytic differentiated HL-60 cells were investigated to elucidate the minimum structure that was required for the activation of their receptor molecules. As we reported previously [12,14], a pentadecapeptide MCT-2 [MCT-2(1–15)] dose-dependently promoted β-hexosaminidase secretion from differentiated HL-60 cells (EC_50_: 20 ± 7 nM, Figure 2 and Table 1), and the maximum response was observed at concentrations greater than 1 μM.

To elucidate the necessity of the N-formyl group for this stimulation, we examined the effects of its removal from MCT-2(1–15). Des-N-formyl MCT-2(1–15) did not induce β-hexosaminidase release, even at a concentration of 100 μM (Figure 2A), demonstrating that the formyl group at the N-terminus of MCT-2(1–15) is crucial for this process.

Next, we examined the importance of the C-terminal sequence of MCT-2(1–15) for the stimulation of β-hexosaminidase release from differentiated HL-60 cells by performing C-terminal truncations. Truncations of one to eight amino acid residues from the C-terminus of MCT-2(1–15) caused consecutive increases in the EC_50_ values without affecting the maximum response. Specifically, the EC_50_ value of MCT-2(1–14) was increased by approximately five-fold compared with that of MCT-2(1–15) (Figure 2B and Table 1). MCT-2(1–13) and MCT-2(1–12) also exhibited decreased activity when compared with MCT-2(1–15), but MCT-2(1–11) had almost the same potency as MCT-2(1–12) (Figure 2B and Table 1). In addition, the activities of MCT-2(1–10), MCT-2(1–9), and MCT-2(1–8) were sequentially attenuated compared with that of MCT-2(1–11), but with the same maximum effect as MCT-2(1–15), and MCT-2(1–7) had almost the same potency as MCT-2(1–8). However, MCT-2(1–6), a derivative that was truncated by nine C-terminal amino acid residues, exhibited a significant decrease of the maximum response compared with MCT-2(1–15) (maximum response: 90 ± 3%) with a 5.7-fold increase in the EC_50_ value compared with MCT-2(1–7). These results indicate that MCT-2(1–7) with an N-formyl group is the minimum structure that is required for maximum stimulation via the activation of FPR2 because the minimum sequence that gave the same level of maximum response as MCT-2(1–15) was MCT-2(1–7), which was also the minimum structure for inducing specific FPR2 activation to cause an increase of [Ca^2+^]_i_ (Figure 1). In addition, the MCT-2(8–15) structure within MCT-2(1–15) may contribute to the binding affinity between MCT-2(1–15) and FPR2, since the removal of one to eight amino acid residues from the C-terminus did not affect the maximum response, but caused a consecutive increase in EC_50_ values (Figure 2B and Table 1).

The effect of C-terminal truncation was examined further because MCT-2(1–6) could still induce β-hexosaminidase release. As a result, MCT-2(1–5), surprisingly, had 6.5-fold higher activity than MCT-2(1–6) (Figure 2C and Table 1) with the same level of maximum response as MCT-2(1–15). Moreover, the activity of MCT-2(1–4) was further reinforced compared with MCT-2(1–5) as well as MCT-2(1–6) (Table 1); nevertheless, the maximum response of MCT-2(1–4) was significantly reduced by approximately 10% compared with MCT-2(1–15). Taken together with the observations that MCT-2(1–5) induced the activation of FPR2 and FPR1, and MCT-2(1–4) specifically activated FPR1 for the increases in [Ca^2+^]_i_ described above (Figure 1), these findings suggest that MCT-2(1–5) effectively promotes the stimulation of β-hexosaminidase release via FPR1 and FPR2 activation. Furthermore, MCT-2(1–4) specifically activates FPR1 to promote stimulation.

### 2.3. Involvement of FPR1 and FPR2 in β-Hexosaminidase Release Stimulated by MCT-2(1–15) and Its Derivatives

**Figure 3:**
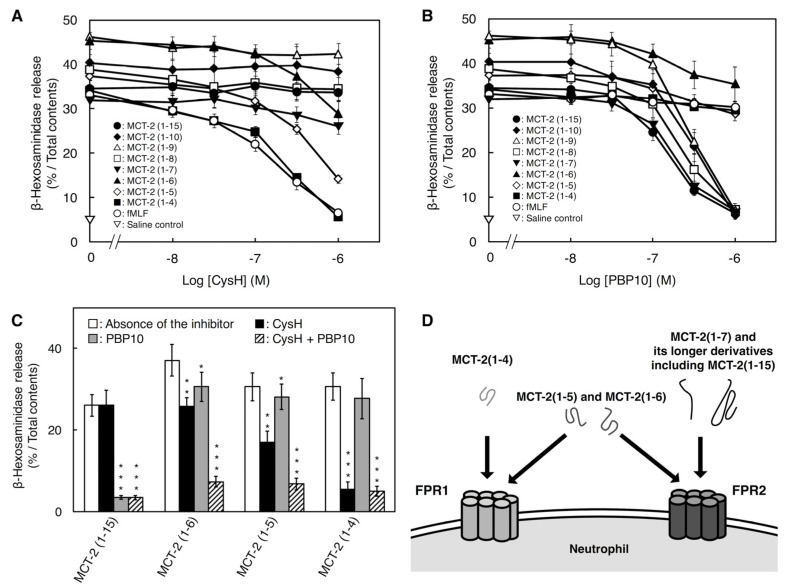
Involvement of FPR1 and FPR2 in β-hexosaminidase release by differentiated HL-60 cells stimulated with MCT-2(1–15) and its derivatives. (**A**,**B**) Concentration-dependent inhibitory effects of inhibitors against FPR1 (CysH) (**A**) or FPR2 (PBP10) (**B**) on β-hexosaminidase release induced by MCT-2(1–15) and its derivatives. (**C**) Inhibitory effects of CysH (1 μM; black), PBP10 (1 μM; gray), or a combination of both inhibitors (1 μM PBP10 and 1 μM CysH; slash lines) on the stimulation of β-hexosaminidase release by MCT-2(1–15) and its derivatives. White bars indicate the stimulation of MCT-2(1–15) and its derivatives in the absence of the inhibitor. The stimulatory concentrations of MCT-2(1–15) and its derivatives were the concentrations that caused a 60% response of the maximum effect. The ability of each peptide in the absence or the presence of the inhibitors was expressed as a percentage of the total enzyme activity, which was the enzyme activity leaked after disruption of the cells with 0.05% Triton X-100 (%/Total). Data are expressed as the mean ± SE of four to six independent experiments. * *p* < 0.05, values significantly different from each peptide in the absence of the inhibitors. (**D**) Receptor preference of MCT-2(1–15) and its derivatives. N-terminal derivatives of MCT-2 with seven amino acid residues [MCT-2(1–7)] or longer than it specifically activate FPR2, whereas MCT-2(1–4) induces the specific activation of FPR1, as well as MCT-2(1–6) and MCT-2(1–5), which activate both FPR1 and FPR2.

To further elucidate the involvement of FPR1 and FPR2 in β-hexosaminidase release from differentiated HL-60 cells stimulated with MCT-2(1–15) and its derivatives, we examined the inhibitory effects of inhibitors against FPR1 [cyclosporin H (CysH)] and FPR2 (PBP10) on this process. Stimulation with MCT-2(1–15) at 40 nM, which induced approximately 60% of the maximum response, was dose-dependently inhibited by PBP10, and 1 μM PBP10 completely inhibited this activity, but 1 μM CysH did not influence MCT-2(1–15)-induced enzyme release (Figure 3A,B). Similarly, the responses induced by MCT-2(1–10), MCT-2(1–9), MCT-2(1–8), and MCT-2(1–7) at a concentration that caused a 60% response of the maximum effect were also abolished in a dose-dependent manner by PBP10 but not CysH (Figure 3A,B). In contrast, stimulation with MCT-2(1–6) and MCT-2(1–5) was partially inhibited by CysH or PBP10, and the combination of inhibitors (1 μM CysH and 1 μM PBP10) almost completely prevented these responses (Figure 3C). Moreover, β-hexosaminidase release induced by MCT-2(1–4) was completely abolished by 1 μM CysH, but was unaffected by 1 μM PBP10 (Figure 3A,B). These results also support the notion that MCT-2(1–7) and its longer N-terminal derivatives induce β-hexosaminidase release from differentiated HL-60 cells via the specific activation of FPR2; in contrast, MCT-2(1–4) specifically activates FPR1, and MCT-2(1–6) and MCT-2(1–5) activate both FPR1 and FPR2 (Figure 3D).

### 2.4. Effects of Substituting Ala for Each Amino Acid Residue of MCT-2(1–15) on β-Hexosaminidase Release by Differentiated HL-60 Cells

**Figure 4:**
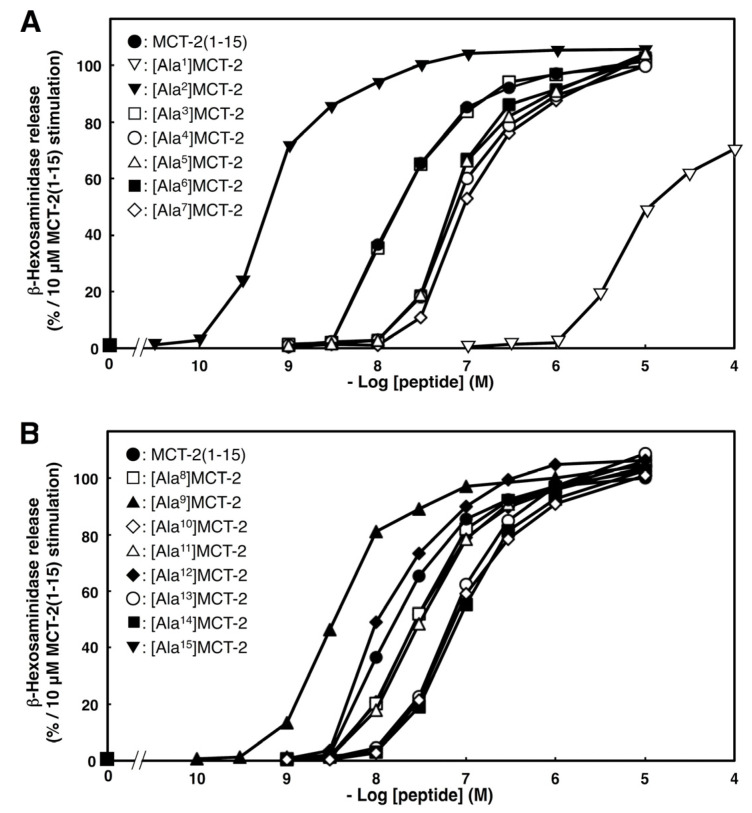
Effects of substituting Ala for each amino acid residue in the MCT-2(1–7) (**A**) or the MCT-2 (8–15) (**B**) sequence within MCT-2(1–15) on β-hexosaminidase release by differentiated HL-60 cells. The differentiated HL-60 cells were stimulated by MCT-2(1–15) or its derivatives at 37 °C for 10 min, and the amount of the released β-hexosaminidase was quantified as described in “Materials and Methods”. The ability of each peptide to cause β-hexosaminidase release is expressed as a percentage of enzyme secretion promoted by 10 μM MCT-2(1-15). Data are expressed as the mean ± SE of four to six independent experiments.

We examined the effect of substituting Ala for each amino acid residue in MCT-2(1–15) on the stimulation of β-hexosaminidase release to elucidate the contribution of each side chain structure to this process. As the MCT-2(1–7) structure with an N-formyl group was indicated as crucial for the activation of FPR2 (Figure 1, Figure 2 and Figure 3), we investigated the effect of substituting Ala for each amino acid residue in positions one to seven within MCT-2(1–15). The replacement of Met^1^ of MCT-2(1–15) with Ala caused a 30% reduction of the maximum response with a dramatic (>500-fold) increase in the EC_50_ value compared with MCT-2(1–15) (Figure 4A and Table 2), indicating that the Met^1^ side chain is important for not only the affinity of MCT-2(1–15) to FPR2, but also for FPR2 activation. The substitution of Met^4^, Arg^5^, Lys^6^, or Ile^7^ with Ala also caused an increase in the EC_50_ value with the same level of maximum response as MCT-2(1–15). In contrast, the replacement of Thr^2^ with Ala promoted a remarkable decrease in the EC_50_ value without affecting the maximum response, although the substitution of Pro^3^ with Ala had no effect on the EC_50_ value. These results suggest that the side chains of Met^4^, Arg^5^, Lys^6^, and Ile^7^, and Thr^2^ within MCT-2(1–15) contribute positively and negatively, respectively, to its affinity to bind to FPR2.

We also examined the effect of substituting Ala for each amino acid residue in the MCT-2(8–15) sequence within MCT-2(1–15) because this structure may be important for the binding affinity between MCT-2(1–15) and FPR2, but not the receptor activation described above. The substitution of Leu^10^, Leu^13^, or Ile^14^ with Ala caused an increase in the EC_50_ value with the same level of maximum response as MCT-2(1–15) (Figure 4B and Table 2). In contrast, the replacement of Pro^9^ with Ala promoted a remarkable decrease in the EC_50_ value without affecting the maximum response [EC_50_: 3 ± 2 nM, Figure 4B and Table 2], although the substitution of Asn^8^, Met^11^, Lys^12^, or Asn^15^ with Ala had no effect. These results suggest that the Leu^10^, Leu^13^, or Ile^14^, and Pro^9^ side chains within MCT-2(1–15) contribute positively and negatively, respectively, to its affinity to bind to FPR2.

### 2.5. Circular Dichroic Spectra of MCT-2(1–15) and Its Derivatives

**Figure 5:**
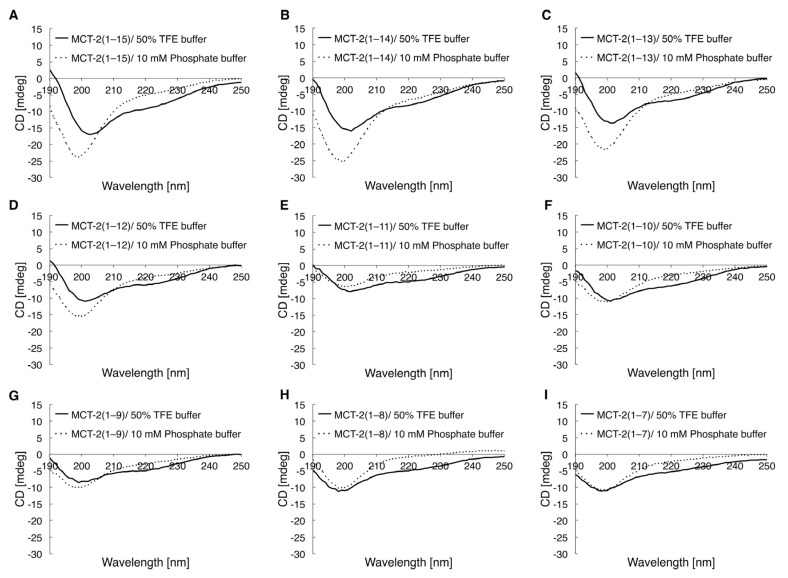
CD spectra of MCT-2(1–15) and its derivatives. The CD spectra of MCT-2(1–15) (**A**), MCT-2(1–14) (**B**), MCT-2(1–13) (**C**), MCT-2(1–12) (**D**), MCT-2(1–11) (**E**), MCT-2(1–10) (**F**), MCT-2(1–9) (**G**), MCT-2(1–8) (**H**), and MCT-2(1–7) (**I**) in 50% TFE buffer (solid lines) or 10 mM phosphate buffer (pH 7.4) (dashed lines) were measured with a CD spectrometer. CD data with peptide solutions (final concentration: 100 μM) were the average of five scans collected at 1 nm intervals. The results are expressed as optical rotation (mdeg).

It was suggested that the Thr^2^, Met^4^, Arg^5^, Lys^6^, Ile^7^, Pro^9^, Leu^10^, Leu^13^, and Ile^14^ side chains within MCT-2(1–15) had an effect on its binding affinity to FPR2 and that the MCT-2(8–15) structure was important for binding to FPR2. Thus, the secondary structures of MCT-2(1–15) and its derivatives were analyzed using circular dichroic (CD) spectra, which is an excellent tool for the rapid investigation of secondary structures. As we reported previously [45], the CD spectrum of MCT-2(1–15) exhibited two minima at 225 nm and 205 nm in TFE solution, suggesting that MCT-2(1–15) predominantly contained an α-helical structure in hydrophilic circumstance (Figure 5A). Similarly, the spectra of the N-terminal MCT-2 derivatives that were truncated by one to six amino acid residues from the C-terminus of MCT-2(1–15) at 100 μM also showed two minima at 225 nm and 202–208 nm in TFE solution, although these minima were consecutively attenuated by the C-terminal truncations (Figure 5B–G). Moreover, the spectra of MCT-2(1–8) and MCT-2(1–7) displayed a minimum at approximately 200 nm in TFE solution (Figure 5H,I), proposing that MCT-2(1–8) and MCT-2(1–7) do not contain defined secondary structures, even under hydrophilic conditions. In contrast, the CD spectra of all of these derivatives including MCT-2(1–15) at 100 μM exhibited a minimum at approximately 200 nm in a hydrophilic phosphate buffer (Figure 5), proposing that MCT-2(1–15) and its derivatives did not form defined secondary structures in hydrophilic conditions.

### 2.6. Time-Dependent Alterations of the Molecular Forms of MCT-2(1–15) in Serum

**Figure 6:**
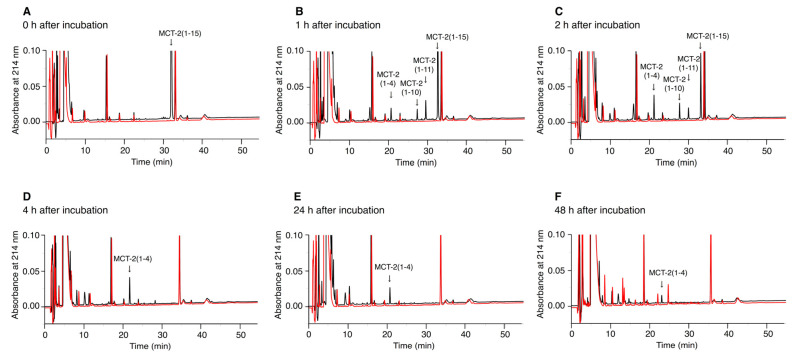
Time-dependent alteration of the molecular forms of MCT-2(1–15) in serum. The alteration of the molecular forms of MCT-2(1–15) in serum was examined as described in the Materials and Methods. Briefly, MCT-2(1–15) was incubated in mouse serum at 37 °C for 0, 1, 2, 4, 24, and 48 h, and samples at those time points were analyzed by RP-HPLC. The panels show the analytical RP-HPLC profile of each sample at 0 (**A**), 1 (**B**), 2 (**C**), 4 (**D**), 24 (**E**), or 48 (**F**) h after incubation. Black and red lines indicate the samples that were incubated with and without MCT-2(1–15), respectively. Analytical conditions: column, 5C_18_ column (4.6 × 150 mm); elution with a linear gradient from 10% to 60% CH_3_CN/0.1% trifluoroacetic acid for 50 min; flow rate, 1 mL/min; detection wavelength, 214 nm. The arrows indicate the peaks containing MCT-2(1–15) or its related peptides that were identified by MALDI-TOF-MS.

We examined the alterations of the molecular forms of MCT-2(1–15) in serum to elucidate the time-dependent degradation of MCT-2(1–15). MCT-2(1–15) was incubated with mouse serum, and the production of MCT-2-derived peptides was analyzed by reverse phase (RP)-HPLC. The molecular masses of the newly produced peaks were measured by MALDI-TOF-mass spectrometry (MS) to identify the molecular forms of the MCT-2-related peptides. As a result, the amount of MCT-2(1–15) in serum was reduced by approximately 50% and 80% at 1 and 2 h after incubation, respectively, and MCT-2(1–15) completely disappeared at 4 h (Figure 6), indicating that its half-life in serum was approximately 1 h. In contrast, MCT-2(1–11), MCT-2(1–10), and MCT-2(1–4) were produced simultaneously with the decrease in the amount of MCT-2(1–15), that is, the production of MCT-2(1–11) and MCT-2(1–10) was detected at 1 h, and the maximum amounts of MCT-2(1–11) and MCT-2(1–10) were observed at 1 and 2 h after incubation, respectively (Figure 6B,C). MCT-2(1–11) and MCT-2(1–10) were not found at 4 h after incubation (Figure 6D). MCT-2(1–4) was detected initially at 1 h after incubation but at a low level, and its maximum level was observed at 4 h (Figure 6B–F). The amount of MCT-2(1–4) was then reduced gradually over time, but it was still present at 48 h (Figure 6F). In addition, no fragmented peptides derived from MCT-2(1–15) were observed at any time points. These results suggest that MCT-2(1–15) is degraded by various proteases in serum, and MCT-2(1–11), MCT-2(1–10), and MCT-2(1–4) are produced simultaneously. Moreover, it is considered that MCT-2(1–4) may be produced by the degradation of MCT-2(1–15), MCT-2(1–11), or MCT-2(1–10), and was present in serum for more than 48 h. These findings suggest that MCT-2(1–15) exerts its functions not only as MCT-2(1–15) but also as MCT-2(1–11), MCT-2(1–10), and MCT-2(1–4).

## 3. Discussion

### 3.1. Recognition Mechanisms of MCT-2(1–15) and Its Derivatives by Formyl-Peptide Receptors

FPR1, which recognizes N-formylated peptides, and its homolog FPR2 are expressed in neutrophils and neutrophilic differentiated HL-60 cells [33,34,35,38,48,49,50,51], and the endogenous pentadecapeptide MCT-2(1–15) specifically activates FPR2 but not FPR1 [36,45] (see also Figure 1, Figure 2 and Figure 3). At first, we examined the structure–activity relationships of MCT-2(1–15) to identify the minimum structure that is required for specific FPR2 activation. We found that the N-formyl group of MCT-2(1–15) was crucial for the induction of β-hexosaminidase release stimulated by the peptide, and suggested that the Met^1^ side chain contributed not only to the affinity of MCT-2(1–15) to bind to FPR2 but also to receptor activation (Figure 1, Figure 2, Figure 3 and Figure 4).

We also showed that MCT-2(1–4), which is an N-terminal tetrapeptide of MCT-2(1–15), induced β-hexosaminidase release, but surprisingly, MCT-2(1–4) specifically activated FPR1 (Figure 1, Figure 2 and Figure 3). Specifically, the evidence that MCT-2(1–4) promoted an increase of [Ca^2+^]_i_ in HEK-293 cells expressing FPR1 but not in those expressing FPR2, as well as the evidence that its stimulation for β-hexosaminidase secretion was specifically inhibited by the FPR1 inhibitor CysH but not FPR2 inhibitor PBP10 indicates that MCT-2(1–4) induces β-hexosaminidase secretion in differentiated HL-60 cells via the specific activation of FPR1. In addition, MCT-2(1–5), a derivative that was extended by C-terminal one amino acid residue to MCT-2(1–4), also exhibited the induction of β-hexosaminidase release (Figure 2C), but in contrast with MCT-2(1–4), MCT-2(1–5) caused an increase of [Ca^2+^]_i_ in both FPR1- and FPR2-expressing HEK-293 cells (Figure 1F,G). These findings suggest that MCT-2(1–5) promotes β-hexosaminidase secretion via the activation of FPR1 and FPR2. The idea that MCT-2(1–5) activates FPR1 and FPR2 to induce β-hexosaminidase secretion was also supported by the use of selective inhibitors against FPR1 and FPR2; the stimulation of MCT-2(1–5) was partially inhibited by CysH or PBP10 and was completely prevented by a combination of both inhibitors (Figure 3C).

In contrast with MCT-2(1–4) and MCT-2(1–5), MCT-2(1–7) induced β-hexosaminidase release via the specific activation of FPR2. Indeed, MCT-2(1–7) promoted β-hexosaminidase secretion in differentiated HL-60 cells and caused an increase of [Ca^2+^]_i_ in HEK-293 cells expressing FPR2 but not in those expressing FPR1 (Figure 1E and Figure 2B). MCT-2(1–7) was also demonstrated to be a full agonist for FPR2 because the maximum response of MCT-2(1–7) to potentiate β-hexosaminidase secretion was at the same level as that induced by MCT-2(1–15), an endogenous specific agonist for FPR2. Moreover, N-terminal derivatives longer than MCT-2(1–7) also exhibited the same maximum effect as MCT-2(1–15) on the stimulation of β-hexosaminidase release, and those EC_50_ values were simultaneously reduced by C-terminal extension, that is, the activity of the derivatives was consecutively potentiated (Figure 2B). In addition, similar to MCT-2(1–7), the N-terminal derivatives MCT-2(1–8), MCT-2(1–9), and MCT-2(1–10) as well as MCT-2(1–15) caused an increase of [Ca^2+^]_i_ only in HEK-293 cells expressing FPR2 (Figure 1A–E). These findings indicate that the N-terminal derivatives longer than MCT-2(1–7) are also specific full agonists for FPR2 to promote β-hexosaminidase secretion. This notion concerning the receptor preference of MCT-2(1–15) and its derivatives was also supported by experiments using selective inhibitors against FPR1 and FPR2. Specifically, the stimulation of β-hexosaminidase release by MCT-2(1–7) and its longer N-terminal derivatives was completely inhibited by the FPR2 inhibitor PBP10, but was unaffected by the FPR1 inhibitor CysH (Figure 3A,B).

MCT-2(1–6) did not induce an increase of [Ca^2+^]_i_ in HEK-293 cells expressing either FPR2 or FPR1, even at 100 μM; nevertheless, it caused β-hexosaminidase secretion (Figure 2). The apparent discrepancy between the induction of β-hexosaminidase release and increase in [Ca^2+^]_i_ stimulated by MCT-2(1–6) may be a result of its weak stimulatory activity for not only FPR1, but also FPR2; the weak stimulation of β-hexosaminidase release by MCT-2(1–6) was presumably a consequence of the slight activation of both FPR1 and FPR2, although this was not evident in HEK-293 cells expressing FPR1 or FPR2. The idea that MCT-2(1–6) weakly activates FPR1 and FPR2 for the induction of β-hexosaminidase secretion was also supported by the use of selective inhibitors against FPR1 or FPR2, i.e., β-hexosaminidase release stimulated by MCT-2(1–6) was partially inhibited by either CysH or PBP10, and was completely prevented by a combination of both inhibitors (Figure 3A–C).

### 3.2. Secondary Structures of MCT-2(1–15) and Its Derivatives for the Interaction with FPR2

In the present study, we showed that the MCT-2(1–7) structure within MCT-2(1–15) is required to induce the maximum response by FPR2 activation. What is the role of the C-terminal MCT-2(8–15) sequence within MCT-2(1–15)? Truncation of one to eight amino acid residues from the C-terminus of MCT-2(1–15) had no effect on the maximum response for the stimulation of β-hexosaminidase release, but did cause a consecutive increase of EC_50_ values (Figure 2B and Table 1), suggesting that the MCT-2(8–15) structure contributes to the binding affinity of MCT-2(1–15) to FPR2, but is not essential for the receptor activation itself. Especially, the removal of the Ile^14^, Leu^13^, Met^11^, and Leu^10^ side chains from MCT-2(1–15) significantly increased the EC_50_ values, suggesting that these hydrophobic amino acid residues are important for the affinity of MCT-2(1–15) with FPR2. Many bioactive peptides form amphipathic α-helical structures when interacting with the cell membrane, and the hydrophobic side chains of those peptides influence their affinity for the cell membrane and their receptors [52,53]. Indeed, MCT-2(1–15) exhibited α-helical signals in CD spectra in hydrophilic conditions, and the truncation of one to eight amino acid residues from its C-terminus caused a simultaneous decrease of the α-helical signals (Figure 5). These findings propose that the α-helical structure of MCT-2(1–15) formed in hydrophobic circumstance may also contribute to its interaction with FPR2. Here, the substitution of Pro^9^ in MCT-2(1–15) with Ala remarkably decreased the EC_50_ value for β-hexosaminidase release, i.e., its activity was potentiated by the exchange (Figure 4B). Taken together with our previous findings that the replacement of Pro^9^ in MCT-2(1–15) with Ala increased the α-helical content in hydrophilic conditions [45], the importance of the amphiphilic α-helical structure of the C-terminal part of MCT-2(1–15) for the improvement of its affinity for FPR2 was also supported by these results.

In this study, the Thr^2^ side chain was also suggested to contribute negatively to the affinity of MCT-2(1–15) for FPR2, that is, the replacement of Thr^2^ in MCT-2(1–15) with Ala promoted a remarkable decrease in the EC_50_ value (Table 2); nevertheless, this substitution did not influence the α-helical content of MCT-2(1–15) [45]. Hence, the increase in activity by its replacement may be a result of an increase in hydrophobicity at position two that improves its binding affinity with FPR2.

### 3.3. Binding Characteristics of MCT-2(1–15) and Its Derivatives to FPR1 and FPR2

**Figure 7:**
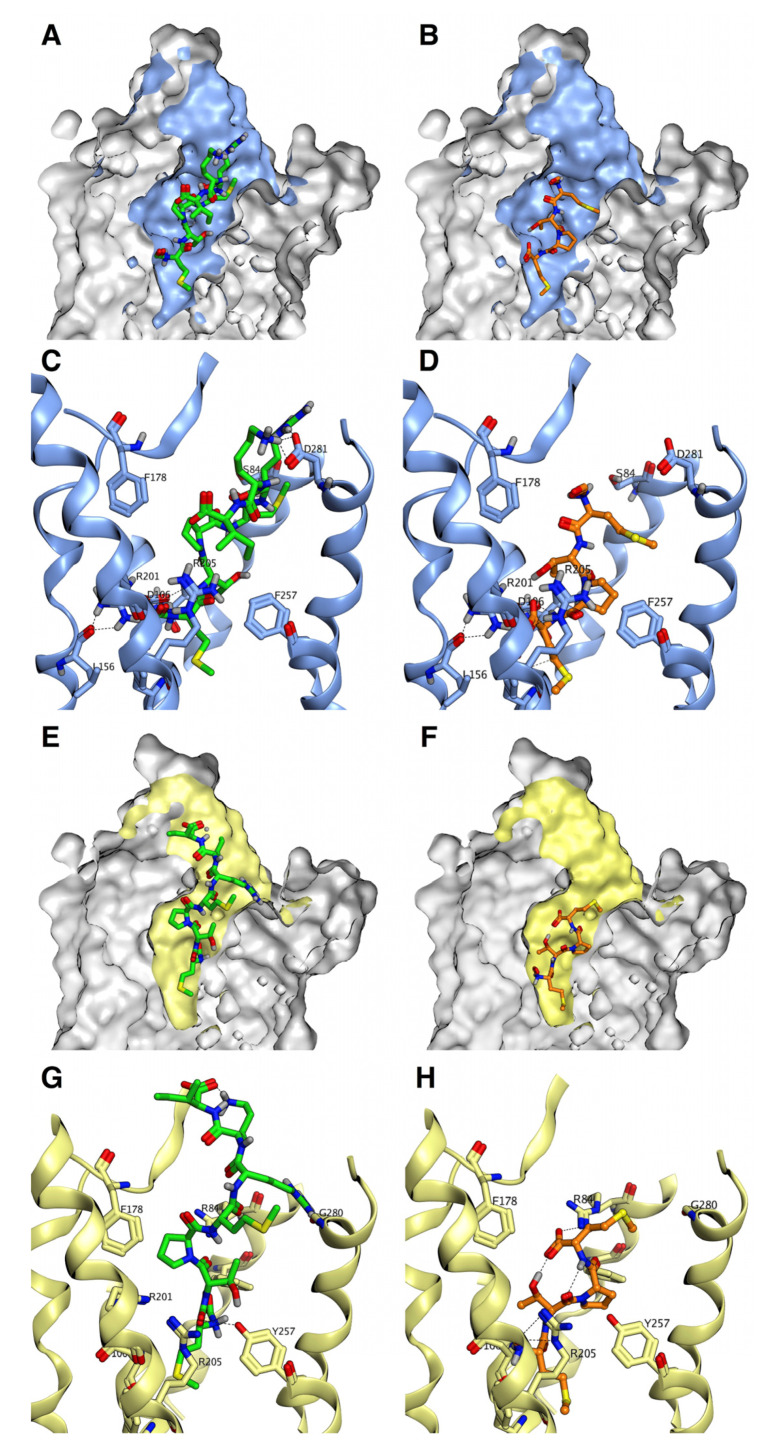
Molecular docking of MCT-2(1–7) and MCT-2(1–4) to FPR2 or FPR1. MCT-2(1–7) and MCT-2(1–4) are shown as green sticks and orange sticks, respectively. (**A**,**B**) The docking model of MCT-2(1–7) (**A**) and MCT-2(1–4) (**B**) to FPR2. (**C**,**D**) Detail of the ligand-binding cavity of FPR2. The structure of the ligand-binding cavity of FPR2 is shown in blue. (**E**,**F**) The docking model of MCT-2(1–7) (**E**) and MCT-2(1–4) (**F**) to FPR1. (**G**,**H**) Detail of the ligand-binding cavity of FPR1. The structure of the ligand-binding cavity of FPR1 is shown in yellow. Hydrogen bonding interactions are shown as dashed lines.

Recently, the tertiary structure of the FPR2-G_i_ complex analyzed by cryo-electron microscopy (EM) has been reported, and that of the FPR1-G_i_ complex was also predicted based on the structure of FPR2 by Zhuang et al. [54]. In addition, formylated peptides containing fMLF were docked to those receptors. These findings suggested that the N-formyl groups of those peptides interacted with the Asp^106^, Arg^201^, and Arg^205^ residues distributed at the bottom of the ligand-binding cavities of FPR1 and FPR2 for the activation of both receptors. Thus, we simulated the docking of MCT-2(1–15) and its derivatives to FPR2 and FPR1 using the Glide program in Schrödinger, which was the program used by Zhuang et al. [54] (Figure 7, Marutani et al., manuscript in preparation). In brief, the N-formyl group of MCT-2(1–15) and its N-terminal derivatives longer than seven amino acid residues were also shown to interact with Asp^106^, Arg^201^, and Arg^205^ of FPR2 but not those of FPR1 because these peptides caused steric hindrance on binding to the cavity of FPR1 (Figure 7A and C vs. Figure 7E,G). Moreover, the Met^1^ side chains of those peptides filled the space at the bottom of the ligand-binding cavity of FPR2 (Figure 7A,C), presumably contributing to the stabilization of the interaction between the N-formyl groups of those peptides and Asp^106^, Arg^201^, and Arg^205^ of FPR2 for receptor activation.

In contrast, MCT-2(1–4) just fit into the binding cavity of FPR1 to promote the interaction between its N-formyl group and Asp^106^, Arg^201^, and Arg^205^ (Figure 7F,H). However, the interaction between MCT-2(1–4) and the ligand-binding cavity of FPR2 was not stabilized due to the lack of hydrophobic and hydrogen bonding interactions as well as the large binding cavity (Figure 7B,D) (docking score: FPR1–MCT-2(1–4) model, −4.84 vs. FPR2–MCT-2(1–4) model, −3.84).

In addition, the Arg^5^ or Lys^6^ residue of MCT-2(1–7) and the C-terminal carboxyl group of MCT-2(1–4) were exhibited to form hydrogen bonds with the Asp^281^ residue of FPR2 and the Arg^84^ residue of FPR1 (Figure 7C,H), respectively, which are distributed at the top of the binding cavities of those receptors, proposing that these hydrogen bonding interactions are of importance for stabilizing further receptor–peptide binding. The present simulation results can well explain those from structure–activity studies of the peptides in the present study in which MCT-2(1–7) and its derivatives longer than seven amino acid residues specifically activated FPR2, whereas MCT-2(1–4) specifically activated FPR1.

### 3.4. Alteration of the Molecular Forms of MCT-2(1–15) in the Bloodstream

Since the receptor preference of MCT-2(1–15) and its derivatives critically depends on the length of its C-terminus, changes in the molecular forms of MCT-2(1–15) in serum were investigated. MCT-2(1–15) in serum was detected within 4 h after incubation, and its half-life was approximately 1 h (Figure 6). In addition, the production of MCT-2-related FPR2 specific agonists MCT-2(1–11) and MCT-2(1–10) was observed at 1 h after incubation, but they were no longer present at 4 h, similar to MCT-2(1–15) (Figure 6). Since MCT-2(1–15) is proposed to be released into the bloodstream from injury tissues as discussed below, these results suggest that MCT-2(1–15) released into the bloodstream initially activates FPR2 for several hours. In contrast, the MCT-2-related FPR1 specific agonist MCT-2(1–4) was found at low levels at 1 h after incubation; its levels then increased gradually over time, and its maximum amount was observed at 4 h. Moreover, MCT-2(1–4) was still present at 48 h (Figure 6). These findings propose that MCT-2(1–15) released into the bloodstream is degraded and the resulting product, MCT-2(1–4), induces the activation of FPR1 following FPR2 activation and continues to activate FPR1 for over 48 h.

### 3.5. Possible Physiological Roles of the Receptor Preference Shift of MCT-2(1–15) from FPR2 to FPR1

The results of this study demonstrate that the receptor preference of MCT-2(1–15) is shifted from FPR2 to FPR1 by the cleavage of its C-terminus. What is the physiological significance of this shift in receptor preference?

FPR1 and FPR2 play critical roles in inflammation including proinflammatory responses, subsequent resolution, and wound healing/tissue regeneration. Specifically, FPR1 and FPR2 are expressed mainly by inflammatory immune cells including neutrophils, monocytes, and monocyte-derived macrophage cells such as tissue-resident macrophages and microglia [35,37,38], and FPR2 is also expressed by a variety of cells including microvascular endothelial cells [38,39]. FPR1 and FPR2 have roles in the mechanisms concerning the infiltration of neutrophils and macrophages into injury sites, and their activation causes various inflammatory responses, including phagocytosis, superoxide generation, and inflammatory cytokine production [35,37,38]. In addition, there is evidence indicating that the activation of FPR2 increases the vascular permeability of endothelial cells [38,39], suggesting a further promotion of neutrophil infiltration from the bloodstream into injury sites following receptor activation in the initial stage of inflammation. Thus, the activation of FPR1 and FPR2 expressed by neutrophils, macrophages, and endothelial cells induces various innate immune responses initiated by the infiltration and activation of neutrophils. Oppositely, it is known that liganded FPR2 suppresses the production of inflammatory cytokines following the acute proinflammatory responses [38,55,56,57,58]. Moreover, FPR1 activation has been demonstrated to promote wound healing/tissue regeneration, including cell proliferation [40,41,42,43,44].

**Figure 8:**
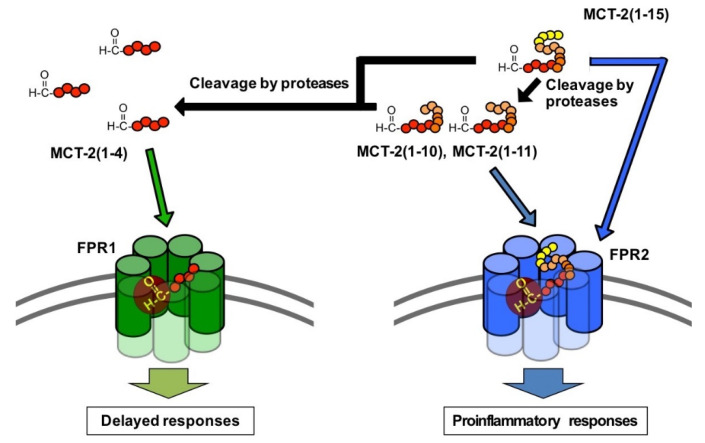
Proposed mechanisms of innate immune responses involving MCT-2(1–15). MCT-2(1–15) released from damaged cells firstly activates FPR2 to induce proinflammatory responses. Released MCT-2(1–15) is then degraded over time to produce MCT-2(1–4), and the resultant MCT-2(1–4) activates FPR1 to promote delayed responses, which may be related to resolution and wound healing/tissue regeneration. See the “Discussion” in detail.

It is believed that mitochondrial-derived N-formylated peptides activate FPR1 and FPR2 as endogenous activating factors. Indeed, mtDAMPs consisting of mitochondria and their contents are released into the bloodstream as a result of sterile tissue damage, such as trauma [15,16,17,18,19,20,21], and still unidentified endogenous N-formylated peptides in mtDAMPs are thought to activate FPR1 and/or FPR2 to induce innate immune responses [15,17,28]. MCT-2(1–15) is the only endogenous N-formylated peptide whose chemical structure has been determined so far, and the presence of MCT-2-related peptides in mtDAMPs was recently observed by immunoblot analysis using a monoclonal antibody against MCT-2 (Tsutsumi et al., unpublished observation). Taken together with these findings, the present results suggest the hypothesis that MCT-2(1–15) is initially released into the bloodstream from damaged cells following tissue injury, and then activates FPR2 specifically to induce acute innate immune responses, including the infiltration and activation of neutrophils (Figure 8). MCT-2(1–15) is then degraded in damaged tissues as well as in the bloodstream over time, and the resulting MCT-2-related FPR1 specific agonist MCT-2(1–4) activates FPR1 to promote delayed responses, which may include resolution and wound healing/tissue regeneration. Indeed, we recently found that a specific neutralizing monoclonal antibody against MCT-2 attenuated the infiltration of neutrophils into injured liver tissue in acetaminophen- or LPS-induced inflammation and prolonged the survival of mice, suggesting that MCT-2(1–15) and its derivatives play a critical role in innate immunity (Takamuro et al., manuscript in preparation). The present findings also indicate the crucial importance of investigating the molecular forms and/or exact chemical structures of those activating factors to elucidate the mechanisms underlying innate immune responses.

## 4. Materials and Methods

### 4.1. Peptides

Human MCT-2 and its derivatives (Table 1 and Table 2) were chemically synthesized by a solid-phase method [59,60,61,62] using a 9-fluorenylmethyloxycarbonyl strategy [12,36]. Synthetic peptides were purified by RP-HPLC on a C_18_ column (20 × 250 mm, COSMOSIL; Nacalai Tesque, Inc., Kyoto, Japan). These peptides were analyzed by RP-HPLC on a C_18_ column (4.6 × 150 mm, COSMOSIL; Nacalai Tesque, Inc.) and were proven to be more than 95% pure (Figures S1 and S2). The molecular weights of the synthesized peptides were also confirmed by MALDI-TOF-MS (Table S1). fMLF was purchased from Nacalai Tesque, Inc.

### 4.2. Preparation of Cells

HEK-293 cells (American Type Culture Collection, Manassas, VA, USA) were maintained in DMEM (Thermo Fisher Scientific, Waltham, MA, USA) containing 10% FBS (Thermo Fisher Scientific) in a humidified atmosphere at 5% CO_2_ and 37 °C. Human acute leukemia-derived HL-60 cells (RIKEN Cell Bank, Ibaraki, Japan) were cultured in RPMI-1640 medium (Thermo Fisher Scientific) containing 10% FBS in a humidified atmosphere at 5% CO_2_ and 37 °C. HL-60 cells were treated with 500 μM dibutyryl-cAMP (Sigma-Aldrich, St. Louis, MO, USA) for 72 h to differentiate the cells into neutrophilic/granulocytic cells as described elsewhere [50].

### 4.3. Establishment of HEK-293 Cells Stably Expressing FPR1 or FPR2

HEK-293 cells stably expressing FPR1 or FPR2 with a Gα_16_ type of G protein were established, as described previously [36]. Briefly, HEK-293 cells were transfected with human FPR1/pcDNA3.1/Zeo or human FPR2/pcDNA3.1/Zeo using Lipofectamine 2000 (Thermo Fisher Scientific). The cells were also co-transfected with Gα_16_/pcDNA3.1/Hygro to induce an agonist-promoted increase in [Ca^2+^]_i_. One cell was placed into each well of a 96-well plate and selected with 100 μg/mL hygromycin (FUJIFILM Wako Pure Chemical, Osaka, Japan) and 250 μg/mL Zeocin (InvivoGen, San Diego, CA, USA). Individual cells were further selected by measuring increases in [Ca^2+^]_i_ stimulated by 1 μM fMLF.

### 4.4. Measurement of [Ca^2+^]_i_

The increase in [Ca^2+^]_i_ stimulated by peptides was assessed, as described previously [11,36]. In brief, HEK-293 cells stably expressing FPR1 or FPR2 with a Gα_16_ type of G protein were washed twice with a HEPES–Na solution (5 mM HEPES, 140 mM NaCl, 4 mM KCl, 1 mM NaH_2_PO_4_, 1 mM MgCl_2_, 1.25 mM CaCl_2_, 11 mM glucose, and 0.2% BSA, pH 7.4). The Ca^2+^-sensitive fluorescence reagent Fura-2-acetoxymethyl ester (Dojin, Kumamoto, Japan) was added to the cell suspension (4 mL; final concentration: 4 μM). The reaction mixture was shielded from light and shaken gently at 37 °C for 60 min to load the cells with Fura-2. The cells were subsequently washed twice with the HEPES–Na solution and a cell suspension was diluted to a final density of 5.0 × 10^5^ cells/mL. The cell suspension (1 mL) was placed into a cuvette and stimulated by peptide solutions (5 μL) with stirring at 37 °C. The ratio of fluorescence intensity at 500 nm by excitation wavelengths of 340 nm and 380 nm was measured using a fluorometer (CAF-100; Japan Spectroscopic Co., Tokyo, Japan).

### 4.5. Assay of β-Hexosaminidase Release

The ability of MCT-2-related peptides to activate HL-60 cells differentiated into neutrophilic/granulocytic cells was evaluated by stimulation of β-hexosaminidase secretion from the cells [63]. Briefly, differentiated HL-60 cells were washed 3 times with ice-cold HEPES-buffered Hank’s solution (HBHS; 10 mM HEPES, 136.9 mM NaCl, 5.4 mM KCl, 1.2 mM CaCl_2_, 0.44 mM KH_2_PO_4_, 0.49 mM MgCl_2_, 0.41 mM MgSO_4_, 0.34 mM Na_2_HPO_4_, 5.5 mM glucose, 4.2 mM NaHCO_3_, and 0.1% BSA, pH 7.4). The cells were resuspended in HBHS at a density of 5.6 × 10^6^ cells/mL, and DNase I (Sigma-Aldrich) and cytochalasin B (Sigma-Aldrich) were each added at a final concentration of 5 μg/mL. The cell suspension in each tube (5.0 × 10^5^ cells/90 μL) was preincubated at 37 °C for 10 min and stimulated with 10 μL synthetic peptide solution at 37 °C for 10 min. The inhibitory effects of inhibitors against FPR1 [CysH (Sigma-Aldrich)] and FPR2 [PBP10 (Tocris Bioscience, Bristol, UK)] on β-hexosaminidase release induced by MCT-2 and its derivatives were also evaluated in the differentiated HL-60 cells; each cell suspension prepared as above (90 μL) was transferred to a tube (5.0 × 10^5^ cells/tube) with 5 μL inhibitor solution for FPR1 (CysH) or FPR2 (PBP10). Each tube was then preincubated at 37 °C for 10 min and stimulated with 5 μL synthetic peptide solution at 37 °C for 10 min. After stimulation, 200 μL ice-cold reaction quenching buffer (25 mM Tris, 123 mM NaCl, and 2.7 mM KCl, pH 7.4) was added to each cell suspension to stop the reaction. Thereafter, these tubes were centrifuged at 4 °C and 2300× *g* for 1 min, and each supernatant was transferred into a new tube.

β-Hexosaminidase activity in the supernatant was measured as described previously [63]. Briefly, 90 μL supernatant was transferred to each well of a 96-well microtiter plate, and 60 μL of a substrate solution for β-hexosaminidase [10 mM p-nitrophenyl N-acetyl-β-D-glucosaminide (Sigma-Aldrich), 40 mM citrate, and 70 mM NaHPO_4_, pH 4.5] was added to initiate the enzyme reaction. After incubation of the plate at 37 °C for 1 h, 100 μL of 400 mM glycine (pH 10.7) was added to stop the reaction. The absorbance at 415 nm for the resulting p-nitrophenol and at 490 nm for the reference was measured using a microtiter plate reader (Viento XS; BioTek Instruments, Winooski, VT, USA).

The ability of each peptide to induce β-hexosaminidase release was expressed as a percentage of enzyme secretion promoted by 10 μM MCT-2(1–15) that induced the maximum response for the elucidation of full or partial agonists to the activity of MCT-2(1–15) (Figure 2 and Figure 4, Table 2) or a percentage of the total enzyme activity, which was the enzyme activity released after disruption of the cells with 0.05% Triton X-100 (Figure 3).

### 4.6. Measurement of Circular Dichroic Spectra

CD spectra of the synthetic peptides were obtained at 25 °C using a J-820 spectrometer (Jasco, Tokyo, Japan) in a quartz cell with a 0.1 cm path length. Spectra were collected between 190 nm and 250 nm with a scan speed of 50 nm/min, response time of 1 s, and bandwidth of 1 nm. Peptide samples with a final concentration of 100 μM were prepared in 10 mM phosphate buffer (pH 7.4) containing 0% or 50% TFE. The baseline scan, which was acquired by measuring the buffer alone, was subtracted from the experimental readings. CD data, which were collected every 1 nm, were the average of 5 scans. The results were expressed as the optical rotation (mdeg).

### 4.7. Analysis of Time-Dependent Alterations of MCT-2 Molecular Forms in Serum

The animal experiments were conducted under the guidance of the Animal Care and Use Committee of the Nagahama Institute of Bio-Science and Technology (Approved No. 047). C57BL/6JJcl mice were purchased from Clea Japan (Tokyo, Japan). All mice were maintained in the Animal Research Facility at the Nagahama Institute of Bio-Science and Technology.

Male C57BL/6JJcl mice, 12–14 weeks of age, weighing 25–30 g, were anesthetized (50 mg/kg pentobarbital), and blood was collected from the caudal vena cava and stored overnight at 4 °C. The blood sample was centrifuged at 4 °C and 20,000× *g* for 20 min, and the supernatant was transferred to a new tube as mouse serum and stored at −80 °C. MCT-2(1–15) was incubated in mouse serum at a final concentration of 500 μM at 37 °C, and aliquots (100 μL) were collected from the incubation mixture after 0, 1, 2, 4, 24, and 48 h. These aliquots were mixed with TCA (final concentration: 3% *w*/*v*) and kept on ice for 30 min to precipitate denatured proteins. The samples were centrifuged at 4 °C and 13,000× *g* for 15 min, and the supernatants were analyzed by RP-HPLC on a C_18_ column (4.6 × 150 mm, Cosmosil; Nacalai Tesque, Inc.). RP-HPLC peaks that contained MCT-2(1–15) and its derivative peptides were analyzed by MALDI-TOF-MS to identify their molecular forms.

### 4.8. Statistical Analysis

Data are expressed as the mean ± standard error (SE) in experiments containing multiple data points. Statistical comparisons between two groups were performed using Student’s *t*-test, and values of *p* < 0.01 were considered significant.

## 5. Conclusions

In the present study, we demonstrated that FPR2 recognizes the MCT-2(1–7) structure with an N-formyl group for its specific activation to induce neutrophilic functions. N-terminal MCT-2 derivatives shorter than seven amino acid residues were shown to lose their specificity for FPR2 and gain the ability to activate FPR1. Moreover, we showed that MCT-2(1–15) was degraded in serum over time, and the MCT-2-related FPR1 specific agonist MCT-2(1–4) was produced, suggesting that the receptor preference of MCT-2(1–15) in the bloodstream is shifted from FPR2 to FPR1 over time by the cleavage of its C-terminus by various proteases. Thus, MCT-2 is proposed to be a factor that controls not only the initiation of innate immune responses against tissue injury, but also delayed responses via the activation of FPR1, which may relate to resolution and wound healing/tissue regeneration. In addition, because the docking simulation of MCT-2(1–15) and its derivatives to FPR1 or FPR2 well explained the receptor-specific activation mechanisms by those peptides as well as the results of structure–activity relationships, the present findings with structural information of FPR2 and FPR1 are expected to accelerate the development of specific antagonists for not only FPR2 but also FPR1 for the treatment of various inflammatory diseases including the recent epidemic of pneumonia that often causes multiple organ failure.

## Figures and Tables

**Table 1:** Amino acid sequences of MCT-2(1–15) and its C-terminal or N-terminal derivatives and their EC_50_ values and maximum effects on the stimulation of β-hexosaminidase release from differentiated HL-60 cells.

Peptide	Sequence																	EC_50_ (nM)	Maximum Effect (%) ^a^
			1	2	3	4	5	6	7	8	9	10	11	12	13	14	15		
MCT-2(1–15)	formyl	-	M	T	P	M	R	K	I	N	P	L	M	K	L	I	N	20.0 ± 3.39	100
MCT-2(1–14)	formyl	-	M	T	P	M	R	K	I	N	P	L	M	K	L	I		96.7 ± 17.6	102
MCT-2(1–13)	formyl	-	M	T	P	M	R	K	I	N	P	L	M	K	L			186 ± 33.6	100 ± 3
MCT-2(1–12)	formyl	-	M	T	P	M	R	K	I	N	P	L	M	K				633 ± 153	98 ± 3
MCT-2(1–11)	formyl	-	M	T	P	M	R	K	I	N	P	L	M					547 ± 93.9	103 ± 3
MCT-2(1–10)	formyl	-	M	T	P	M	R	K	I	N	P	L						1800 ± 173	98 ± 3
MCT-2(1–9)	formyl	-	M	T	P	M	R	K	I	N	P							10,760 ± 850	104 ± 3
MCT-2(1–8)	formyl	-	M	T	P	M	R	K	I	N								41,333 ± 2517	100 ± 2
MCT-2(1–7)	formyl	-	M	T	P	M	R	K	I									35,500 ± 500	101 ± 2
MCT-2(1–6)	formyl	-	M	T	P	M	R	K										202,667 ± 2309	90 ± 2 **
MCT-2(1–5)	formyl	-	M	T	P	M	R											26,000 ± 1581	104 ± 2
MCT-2(1–4)	formyl	-	M	T	P	M												18,000 ± 1323	88 ± 2 ***
Des-*N*-formyl MCT-2			M	T	P	M	R	K	I	N	P	L	M	K	L	I	N	>1,000,000	0

^a^ The ability of each peptide to cause β-hexosaminidase release is expressed as a percentage of enzyme secretion promoted by 10 μM MCT-2(1–15). Data are expressed as the mean ± SE of four to six independent experiments. ** *p* < 0.01; *** *p* < 0.001, values significantly different from MCT-2(1–15).

**Table 2:** Amino acid sequences of MCT-2(1–15) and its Ala-substituted derivatives and their EC_50_ values and maximum effects on the induction of β-hexosaminidase release from differentiated HL-60 cells.

Peptide	Sequence																	EC_50_ (nM)	Maximum Effect (%) ^a^
			1	2	3	4	5	6	7	8	9	10	11	12	13	14	15		
MCT-2(1–15)	formyl	-	M	T	P	M	R	K	I	N	P	L	M	K	L	I	N	20.0 ± 3.39	100
[Ala^1^]MCT-2	formyl	-	A	T	P	M	R	K	I	N	P	L	M	K	L	I	N	>10,000	70 ± 2 ***
[Ala^2^]MCT-2	formyl	-	M	A	P	M	R	K	I	N	P	L	M	K	L	I	N	0.61 ± 0.07	106 ± 2
[Ala^3^]MCT-2	formyl	-	M	T	A	M	R	K	I	N	P	L	M	K	L	I	N	16.3 ± 0.29	102 ± 1
[Ala^4^]MCT-2	formyl	-	M	T	P	A	R	K	I	N	P	L	M	K	L	I	N	68.0 ± 2.65	100 ± 2
[Ala^5^]MCT-2	formyl	-	M	T	P	M	A	K	I	N	P	L	M	K	L	I	N	61.3 ± 0.58	104 ± 1
[Ala^6^]MCT-2	formyl	-	M	T	P	M	R	A	I	N	P	L	M	K	L	I	N	64.0 ± 3.00	103 ± 2
[Ala^7^]MCT-2	formyl	-	M	T	P	M	R	K	A	N	P	L	M	K	L	I	N	94.7 ± 3.21	104 ± 2
[Ala^8^]MCT-2	formyl	-	M	T	P	M	R	K	I	A	P	L	M	K	L	I	N	31.0 ± 3.97	106 ± 1
[Ala^9^]MCT-2	formyl	-	M	T	P	M	R	K	I	N	A	L	M	K	L	I	N	3.02 ± 1.21	105 ± 1
[Ala^10^]MCT-2	formyl	-	M	T	P	M	R	K	I	N	P	A	M	K	L	I	N	73.0 ± 13.3	100 ± 2
[Ala^11^]MCT-2	formyl	-	M	T	P	M	R	K	I	N	P	L	A	K	L	I	N	36.7 ± 11.0	102 ± 1
[Ala^12^]MCT-2	formyl	-	M	T	P	M	R	K	I	N	P	L	M	A	L	I	N	12.3 ± 0.29	106 ± 3
[Ala^13^]MCT-2	formyl	-	M	T	P	M	R	K	I	N	P	L	M	K	A	I	N	83.7 ± 3.54	107 ± 3
[Ala^14^]MCT-2	formyl	-	M	T	P	M	R	K	I	N	P	L	M	K	L	A	N	76.7 ± 11.9	103 ± 3
[Ala^15^]MCT-2	formyl	-	M	T	P	M	R	K	I	N	P	L	M	K	L	I	A	29.3 ± 1.49	104 ± 3

^a^ The ability of each peptide to cause β-hexosaminidase release is expressed as a percentage of enzyme secretion promoted by 10 μM MCT-2. Data are expressed as the mean ± SE of four to six independent experiments. *** *p* < 0.001, values significantly different from MCT-2(1–15).

## Data Availability

All data and materials presented in this article and in the supplementary information are available from the corresponding author upon reasonable request.

## Supplementary Materials

The following are available online at https://www.mdpi.com/article/10.3390/ijms22084084/s1.

## Abbreviations

[Ca^2+^]_i_	Concentration of intracellular free Ca^2+^
CD	Circular dichroic
CysH	Cyclosporin H
EM	Electron microscopy
fMLF	Formyl-Met-Leu-Phe
FPR1	Formyl peptide receptor 1
FPR2	Formyl peptide receptor 2
HBHS	HEPES-buffered Hank’s solution
MCT-2	Mitocryptide-2
MS	Mass spectrometry
mtDAMPs	Mitochondrial damage-associated molecular patterns
RP	Reverse phase
TFE	2,2,2-trifluoroethanol
