# Supplementary material for: Mitocryptide-2: Identification of Its Minimum Structure for Specific Activation of FPR2–Possible Receptor Switching from FPR2 to FPR1 by Its Physiological C-terminal Cleavages
Source: Int J Mol Sci. 2021 Apr 15;22(8):4084. doi: 10.3390/ijms22084084 (PMC8071274; doi:10.3390/ijms22084084)
Supplement: Supplementary file 1 [file ijms-22-04084-s001.zip › Supplementary Material .pdf]

Supplementary Material

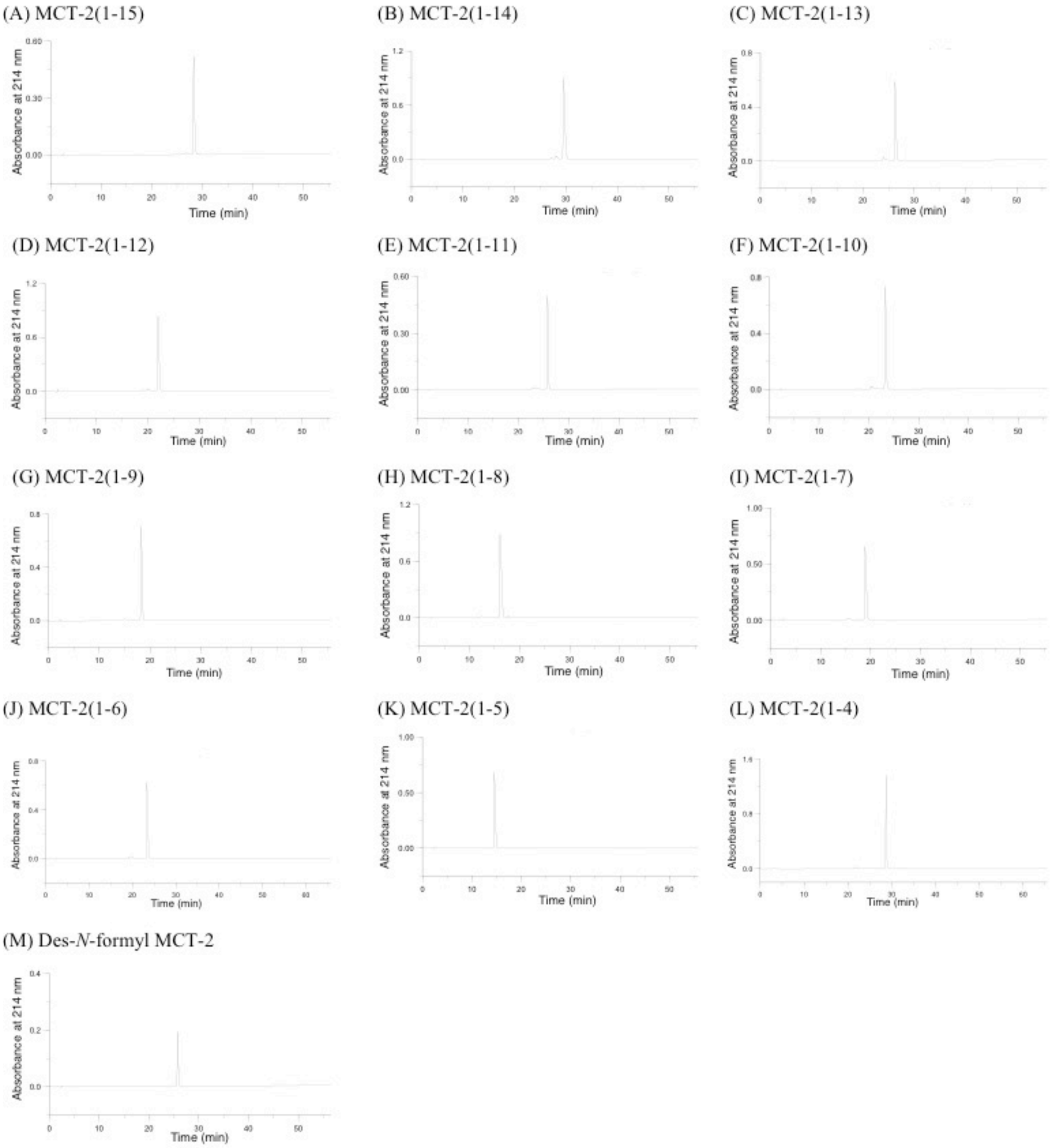

Figure S1. Analytical RP-HPLC profiles of MCT-2(1-15) (A), MCT-2(1-14) (B), MCT-2(1-13) (C), MCT-2(1-12) (D), MCT-2(1-11) (E), MCT-2(1-10) (F), MCT-2(1-9) (G), MCT-2(1-8) (H), MCT-2(1-7) (I), MCT-2(1-6) (J), MCT-2(1-5) (K), MCT-2(1-4) (L), and Des-N-formyl MCT-2 (M).
Analytical conditions: column, $5C_{18}$ column (4.6 × 150 mm); elution with a linear gradient from 10% to 60% CH_3CN/0.1% trifluoroacetic acid for 50 min (A–I, K and M) or 0% to 60% CH_3CN/0.1% trifluoroacetic acid for 60 min (J and L); flow rate, 1 mL/min; detection wavelength, 214 nm.

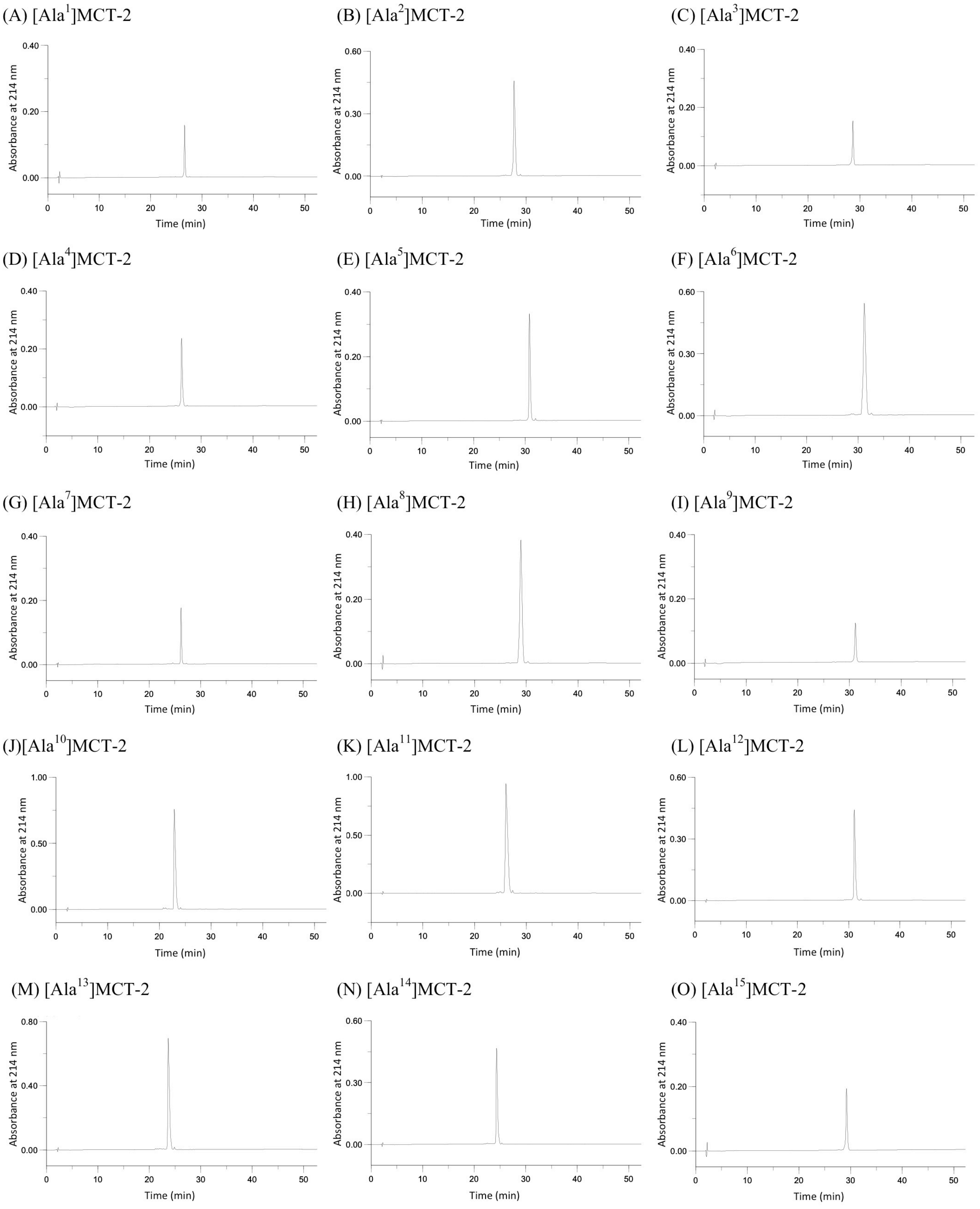

Figure S2. Analytical RP-HPLC profiles of [Ala^{1}]MCT-2 (A), [Ala^{2}]MCT-2 (B), [Ala^{3}]MCT-2 (C), [Ala^{4}]MCT-2 (D), [Ala^{5}]MCT-2 (E), [Ala^{6}]MCT-2 (F), [Ala^{7}]MCT-2 (G), [Ala^{8}]MCT-2 (H), [Ala^{9}]MCT-2 (I), [Ala^{10}]MCT-2 (J), [Ala^{11}]MCT-2 (K), [Ala^{12}]MCT-2 (L), [Ala^{13}]MCT-2 (M), [Ala^{14}]MCT-2 (N), and [Ala^{15}]MCT-2 (O).

Analytical conditions: column, 5C_{18} column (4.6 × 150 mm); elution with a linear gradient from 10% to 60% CH_3CN/0.1% trifluoroacetic acid for 50 min; flow rate, 1 mL/min; detection wavelength, 214 nm.

Supplementary Table 1. Analytical data by MALDI-TOF-MS for MCT-2(1-15) and its derivatives.

Peptide	MALDI-TOF-MS (m/z, $[M+H]^+$)	
	Observed	Calculated
MCT-2(1-15)	1827.81	1828.03
MCT-2(1-14)	1713.61	1713.98
MCT-2(1-13)	1600.88	1600.88
MCT-2(1-12)	1487.7	1487.79
MCT-2(1-11)	1359.69	1359.72
MCT-2(1-10)	1228.60	1228.70
MCT-2(1-9)	1115.55	1115.58
MCT-2(1-8)	1018.56	1018.52
MCT-2(1-7)	904.42	904.49
MCT-2(1-6)	791.36	791.40
MCT-2(1-5)	663.35	663.31
MCT-2(1-4)	529.19[a]	507.21
Des-*N*-formyl MCT-2	1799.99	1800.01
[Ala^{1}]MCT-2	1769.27	1769.20
[Ala^{2}]MCT-2	1799.75	1799.29
[Ala^{3}]MCT-2	1803.24	1803.28
[Ala^{4}]MCT-2	1769.21	1769.21
[Ala^{5}]MCT-2	1743.97	1744.21
[Ala^{6}]MCT-2	1772.75	1772.22
[Ala^{7}]MCT-2	1787.51	1787.24
[Ala^{8}]MCT-2	1786.55	1786.29
[Ala^{9}]MCT-2	1803.38	1803.28
[Ala^{10}]MCT-2	1787.36	1787.24
[Ala^{11}]MCT-2	1769.47	1769.21
[Ala^{12}]MCT-2	1772.35	1772.22
[Ala^{13}]MCT-2	1787.60	1787.24
[Ala^{14}]MCT-2	1787.81	1787.24
[Ala^{15}]MCT-2	1786.98	1786.29

[a]Signal corresponding to a sodiated peptide ($[M+Na]^+$).
